# Dual targeting of HSP70 does not induce the heat shock response and synergistically reduces cell viability in muscle invasive bladder cancer

**DOI:** 10.18632/oncotarget.26021

**Published:** 2018-08-24

**Authors:** Thomas Prince, Andrew Ackerman, Alice Cavanaugh, Brielle Schreiter, Brendon Juengst, Chaylen Andolino, John Danella, Mitch Chernin, Heinric Williams

**Affiliations:** ^1^ Urology Department, Geisinger Clinic, Danville, 17822 PA, USA; ^2^ Weis Center for Research, Geisinger Clinic, Danville, 17822 PA, USA; ^3^ Biology Department, Bucknell University, Lewisburg, 17837 PA, USA

**Keywords:** HSP70, HSP90, bladder cancer, drug combination, synergy

## Abstract

Muscle invasive bladder cancer (MIBC) is a common malignancy and major cause of morbidity worldwide. Over the last decade mortality rates for MIBC have not decreased as compared to other cancers indicating a need for novel strategies. The molecular chaperones HSP70 and HSP90 fold and maintain the 3-dimensional structures of numerous client proteins that signal for cancer cell growth and survival. Inhibition of HSP70 or HSP90 results in client protein degradation and associated oncogenic signaling. Here we targeted HSP70 and HSP90 with small molecule inhibitors that trap or block each chaperone in a low client-affinity “open” conformation. HSP70 inhibitors, VER155008 (VER) and MAL3-101 (MAL), along with HSP90 inhibitor, STA-9090 (STA), were tested alone and in combination for their ability to reduce cell viability and alter protein levels in 4 MIBC cell lines. When combined, VER+MAL synergistically reduced cell viability in each MIBC cell line while not inducing expression of heat shock proteins (HSPs). STA+MAL also synergistically reduced cell viability in each cell line but induced expression of cytoprotective HSPs indicating the merits of targeting HSP70 with VER+MAL. Additionally, we observed that STA induced the expression of the stress-related transcription factor HSF2 while reducing levels of the co-chaperone TTI1.

## INTRODUCTION

Bladder cancer is the fifth most common cancer accounting for 4.6% of new cancer cases in the United States [1]. While the number of new cases have been declining by 0.8% annually from 2004 to 2013, the mortality rates over this period has been stable [2]. Urothelial (transitional cell) carcinoma represents over 90% of malignant histological subtypes of bladder cancer [3]. Clinically, it may present as non-muscle invasive disease bladder cancer, muscle invasive bladder cancer (MIBC) or metastatic disease [4]. Most individuals die from overt metastatic disease or progression of MIBC to metastatic disease. Since there is no cure for metastatic disease, management is directed toward controlling the cancer while confined within the organ [4, 5]. Once metastasized, cisplatin based combination chemotherapy remains the mainstay for managing advanced MIBC [6].

An integrated genomic study of MIBC clinical samples that looked at tumor specific alterations at the level of recurrent somatic mutations, copy number variations, gene translocations, and changes in both mRNA and miRNA expression identified several key oncogenic signaling pathways. These pathways included cell cycle control (93% of cases), histone modification (89%), kinase-PIK3CA signaling (72%) and nucleosome remodeling (64%) [7]. Together these data highlighted the genomic heterogeneity of MIBC in individuals presenting with the same transitional cell carcinoma histological subtype. It also suggested that multiple pathways may be actively involved in the disease process and thus will likely need to be simultaneously targeted for improved therapeutic outcomes. Earlier strategies focused on targeting single components within an oncogenic pathway have only been marginally successful, suggesting the need to identify therapeutic targets that can disrupt multiple overlapping survival and growth pathways utilized by cancer.

In this regard, certain families of heat shock proteins (HSPs) have shown promise as therapeutic targets due to their roles as molecular chaperones. HSPs act as molecular chaperones folding and maintaining the 3-dimensional structure of other proteins often referred to as “clients” [8]. This chaperone activity provides cells essential protein-folding capacity, which helps maintain proteostasis [9]. When cells experience stress, protein-folding capacity is increased through the expression of inducible HSPs. Cancer cells exist in toxic microenvironments and must hijack the cytoprotective heat shock response to increase their protein-folding capacity to survive [10, 11]. Small molecule drugs that inhibit HSP chaperone activity reduce this capacity and disrupt proteostasis –pushing cancer cells to self-destruct [12].

The nuclear-cytosolic 90-kDa heat shock protein, HSP90, is collectively composed of two paralogs: stress inducible Hsp90α and constitutively expressed Hsp90β. Together they fold and maintain a myriad of components involved in promoting cancer cell growth and survival [13]. Often referred to as the “signal transduction chaperone” [14], HSP90 is estimated to chaperone 60% kinases, 30% transcription factors and 7% of E3-ligases [15]. Inhibitors that target the N-terminal ATPase-domain of HSP90 trap it in a low-affinity “open” conformation that induces degradation of client proteins. This results in disruption of multiple oncogenic signaling pathways [16, 17]. Due to this ability, several N-terminal HSP90 inhibitors have been tested in the cancer clinic as monotherapies, however, none have shown enough effectiveness to clear phase III trials [18]. This lack of efficacy is likely related to a concomitant activation of the stress response and resulting increase in HSP70 expression. Another molecular chaperone with high protein-folding capacity, HSP70 compensates for the loss of HSP90 activity and maintains pro-survival signaling pathways thereby enabling an escape mechanism for cancer cells [19, 20].

In humans, the HSP70 family includes 8 members with stress-inducible Hsp70-1 being the most studied in relation to cancer. Elevated levels of Hsp70-1 are associated with a variety of malignancies where it enhances cell growth, suppresses senescence and confers resistance to cytostatic drugs and radiation therapy [21, 22]. In MIBC, Hsp70-1 overexpression correlates with increased clinical stage, tumor grade and poor patient outcome [23]. Inhibiting collective HSP70 protein-folding capacity may be a rational alternative to targeting HSP90 [24]. Previous studies have shown that inhibiting or depleting HSP70 does not induce a robust stress response that increases total HSP levels [25, 26]. Furthermore, HSP70 is a more promiscuous chaperone that operates independently and upstream of HSP90 activity [27]. Thus, many HSP90 clients first encounter HSP70 before being handed off to Hsp90 for further folding.

**Figure 1:**
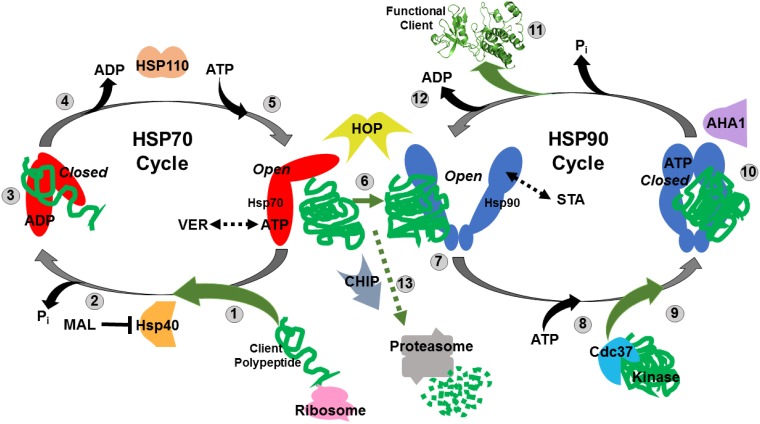
Simplified model of the Hsp70 and Hsp90 chaperone cycles (**1**) Nascent polypeptides emerging from translating ribosomes are recognized by Hsp40 and associate with ATP-bound HSP70 in an “open” conformation. VER155008 (VER) mimics ATP and maintains HSP70 in an “halfway-open” conformation. (**2**) HSP40 induces HSP70 ATPase activity releasing inorganic phosphate and causing Hsp70 to switch to a “closed” conformation that tightly binds peptides. MAL3-101 (MAL) prevents this switch by blocking HSP40-HSP70 interaction. (**3**) Hydrophobic amino acid stretches within the client polypeptides are bound and protected by the substrate binding domain of HSP70 in a “closed” conformation allowing the peptide to fold and not aggregate. (**4**) ADP is exchanged for ATP by HSP70 nucleotide exchange factors such as HSP110. (**5**) ATP-bound HSP70 adopts “open” conformation to allow for client polypeptides release. (**6**) Transfer of the client polypeptides from HSP70 to HSP90 dimer is coordinated by HOP. (**7**) Partially folded clients associate with HSP90 in its “open” conformation, STA-9090 (STA) locks HSP90 in a similar “open” conformation. (**8**) ATP binds to the N-terminal domain of HSP90. (**9**) Protein kinase clients may be loaded on to HSP90 by Cdc37 for folding at this stage. (**10**) HSP90 ATPase activity initiated by AHA1 induces structural rearrangements that result in a “closed” conformation that strongly binds the client proteins and further promote folding. (**11**) Release of folded, functional client protein with oncogenic potential. (**12**) ADP is released allowing the HSP90 cycle to reset and load client polypeptides. (**13**) Oncogenic client proteins that associate with the “open” conformations of HSP70 and HSP90 for extended periods due drug inhibition may be ubiquitinated by the E3-ligase CHIP and directed to the proteasome for destruction.

HSP70 and HSP90 along with a host of co-chaperones, cooperate to fold newly synthesized polypeptides and denatured proteins by linking together their ATP-fueled chaperone cycles. This complexity provides opportunities to reduce protein-folding capacity by blocking ATP-binding, ADP-exchange or key protein-protein interactions that drive the chaperone cycle with small molecule inhibitors (Figure 1). VER155008 (VER, MW 556.4) is an ATP analogue that occupies the Hsp70 nucleotide-binding pocket and traps HSP70 in a “halfway-open” conformation that prevents active chaperone function [28]. VER has been shown to reduce proliferation in breast, colon and lung cancer cell lines [29, 30]. MAL3-101 (MAL, MW 931.14), an allosteric inhibitor, inhibits HSP70 ATPase activity by blocking Hsp40 co-chaperone interaction [31]. This extends the time HSP70 remains in an “open” conformation and unable to tightly bind client proteins. MAL has shown effectiveness in multiple myeloma and Merkel cell carcinoma models [32, 33]. STA9090 (STA, ganetespib, MW 364.4) is an N-terminal HSP90 inhibitor that has been extensively studied in both the laboratory and cancer clinic [18].

Previous work in our lab showed that combining STA with VER synergistically reduced cell viability and disrupted oncogenic signaling in 4 different MIBC cell lines [34]. The STA+VER combination prevented active chaperone function by holding both HSP90 and HSP70 in “open” conformations. This reduced total protein-folding capacity and blunted increased HSP70 activity induced by STA.

In this report we compare combinations of STA+VER, STA+MAL and VER+MAL along with each single agent for their ability to reduce cell viability, degrade oncogenic clients and modulate the stress response. We also test STA+MAL and VER+MAL for their ability to synergistically reduce cell viability in 4 MIBC cell lines. Our findings indicate that despite requiring elevated concentrations, dual targeting of HSP70 with VER+MAL synergistically kills MIBC cells and disrupts oncogenic signaling while not stimulating the heat shock response. Furthermore, we found that STA induces the expression of the stress response transcription factor, HSF2, while reducing the expression of the co-chaperone TTI1.

## RESULTS

### Effects of targeting HSP70 and HSP90 on cell viability

**Figure 2:**
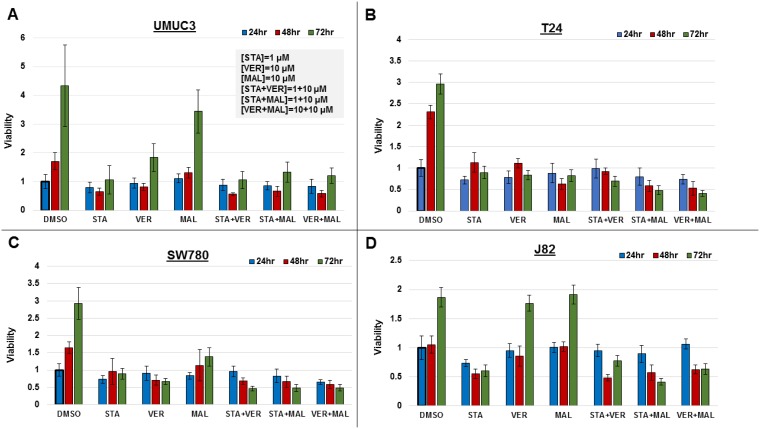
Combinations of HSP70 and HSP90 inhibitors reduce cell viability to different degrees over time MIBC cell lines (**A**) UMUC3, (**B**) T24, (**C**) SW780 and (**D**) J82 were plated and treated with STA, VER and MAL alone or in combination for 24, 48, and 72 hours. Cell viability was determined by crystal violet and normalized to the 24-hour DMSO control for each cell line ± standard deviation. Statistical analysis of results for each cell line at each timepoint and across timepoints are found in Supplementary Figures 2–4.

To directly compare the efficacy of each drug and combination, we assayed for their effect on cell viability in MIBC cell lines, UMUC3, T24, SW780 and J82, over a 72-hour time course. Cells were seeded in 96-well plates and incubated with the 1 μM STA, 10 μM VER, 10 μM MAL, STA+VER [1 + 10 μM], STA+MAL [1 + 10 μM], VER+MAL [10 + 10 μM] or vehicle DMSO for 24, 48 or 72 hours. Plates were harvested and assayed for cell viability by resazurin salt reduction, which tests for aerobic respiration [35], followed by crystal violet incorporation, which tests for DNA-protein content [36]. Both assays produced similar results. Data from the crystal violet assays are shown in Figure 2, while data from the resazurin assays are in Supplementary Figure 1.

Each alone drug and in combination was effective to a different degree in each cell line. In UMUC3 cells, 1 μM STA was as effective at inhibiting cell growth as each of the combinations. In T24 cells, STA+MAL and VER+MAL were most effective at total drug concentrations of 11 μM and 20 μM, respectively. Similarly, in SW780 cells, STA+MAL and VER+MAL were most effective. In J82 cells, STA+MAL was most effective.

Drug treatments that significantly reduced cell viability from the 24-hour to the 72-hour time points were considered cytotoxic. Only VER in SW780 and STA in J82 cells were observed to be cytotoxic as monotherapy. Fortunately, combinations of STA-VER, STA+MAL and VER+MAL were observed to be cytotoxic in T24 and SW780 cells, while STA+MAL and VER+MAL were cytotoxic in J82 cells. Statistical analysis of each treatment across each time point are given in Supplementary Figure 2.

Drug treatments that reduced cell viability at 72 hours as compared to DMSO control suggested cytostatic or anti-proliferative effects. Most monotherapies and combinations were cytostatic at 72 hours, with the exceptions of MAL and VER in J82 cells and MAL in UMUC3 cells. In these instances, VER or MAL did not significantly inhibit proliferation compared to DMSO control suggesting drug resistance. Also concerning when checking for higher 72-hour to 24-hour viability levels, all mono-therapies and combinations in at least one cell line showed signs of drug resistance at the described concentrations. Statistical analysis of each cell line at each timepoint are given in Supplementary Figures 3 and 4.

These results indicate that each MIBC cell line responds differently to targeting HSP70 and/or HSP90. This likely reflects the degree of dependency of each cell line on HSP70 and HSP90 chaperone activity, drug resistance mechanisms and the potency of each drug. Dual targeting of HSP70 with VER+MAL was effective at reducing cell viability and was cytotoxic in 3 of the 4 cell lines tested.

### Dual targeting of HSP70 or combined targeting of HSP90 and HSP70 synergistically reduce MIBC cell viability

**Figure 3:**
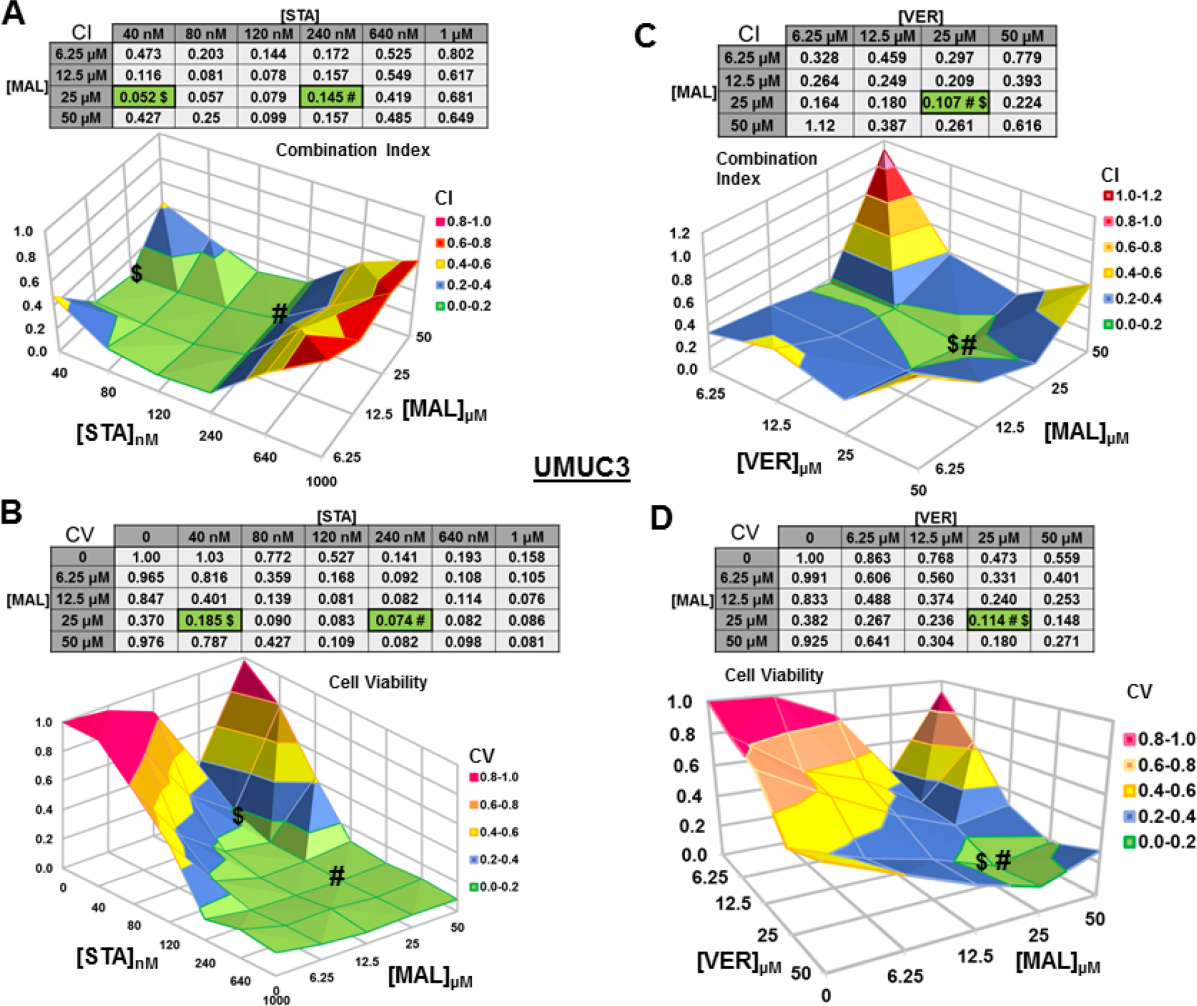
Combinations of HSP70 and HSP90 inhibitors synergistically reduce UMUC3 cell viability UMUC3 cells were treated for 48 hours with a range of STA+MAL or VER+MAL concentration combinations. Cell viability (CV) was determined by crystal violet and normalized to DMSO control for each treatment. Combination Indices (CI) were calculated by CompuSyn. CI-values less than 1.0 indicated the concentration combination was synergistic at reducing cell viability. (**A**) STA+MAL surface graph and CI-values, (**B**) STA+MAL surface graph and CV-values, (**C**) VER+MAL surface graph and CI-values and (**D**) VER+MAL surface graph and CV-values. $ and # indicate the lowest CI-value and CV-value for each treatment for each treatment, respectively, each value in the table is colored according to its position on the surface graph.

**Figure 4:**
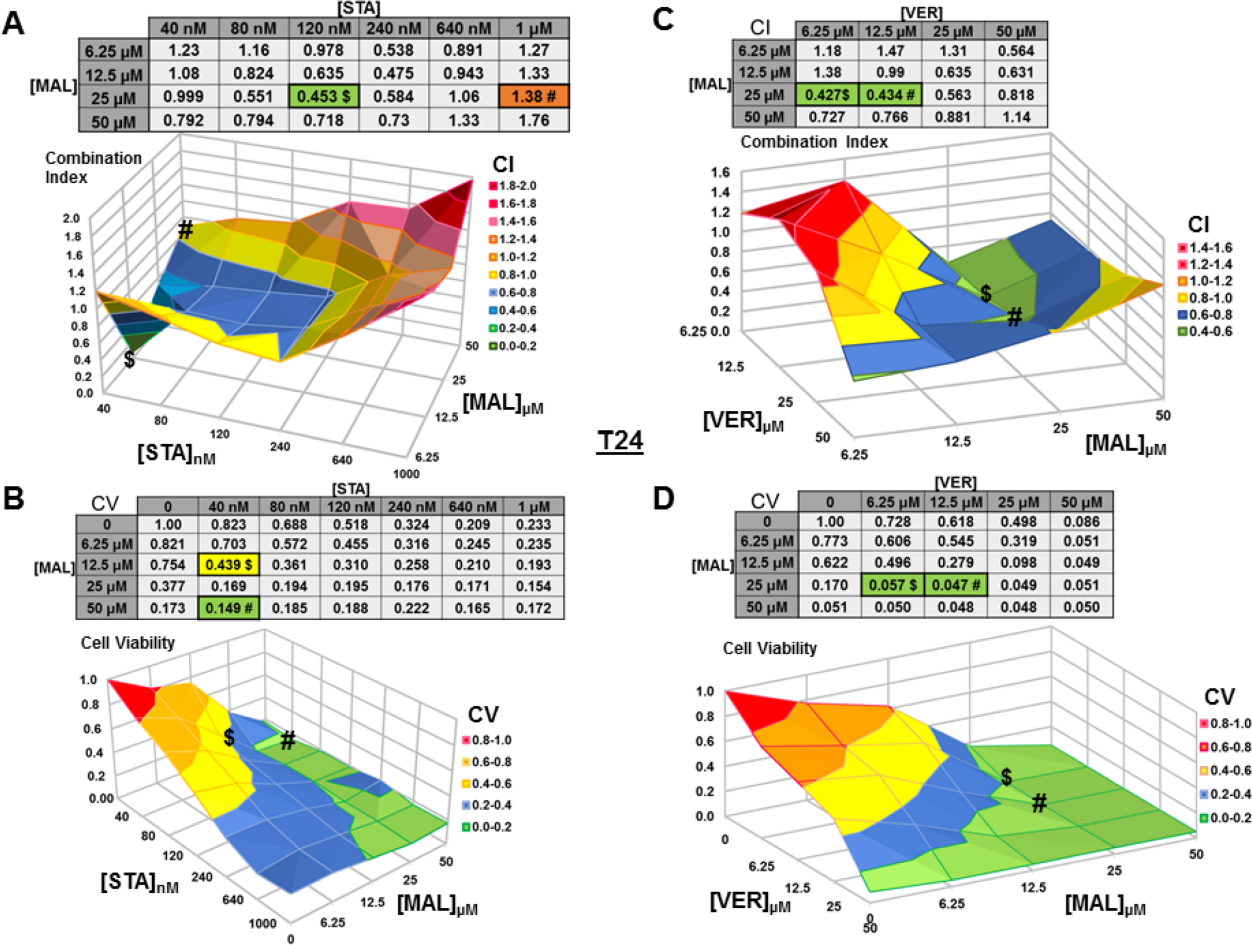
Combinations of HSP70 and HSP90 inhibitors synergistically reduce T24 cell viability T24 cells were treated for 48 hours with a range of STA+MAL or VER+MAL concentration combinations. (**A**) STA+MAL surface graph and CI-values, (**B**) STA+MAL surface graph and CV-values, (**C**) VER+MAL surface graph and CI-values and (**D**) VER+MAL surface graph and CV-values. $ and # indicate the lowest CI-value and CV-value for each treatment for each treatment, respectively, each value in the table is colored according to its position on the surface graph.

**Figure 5:**
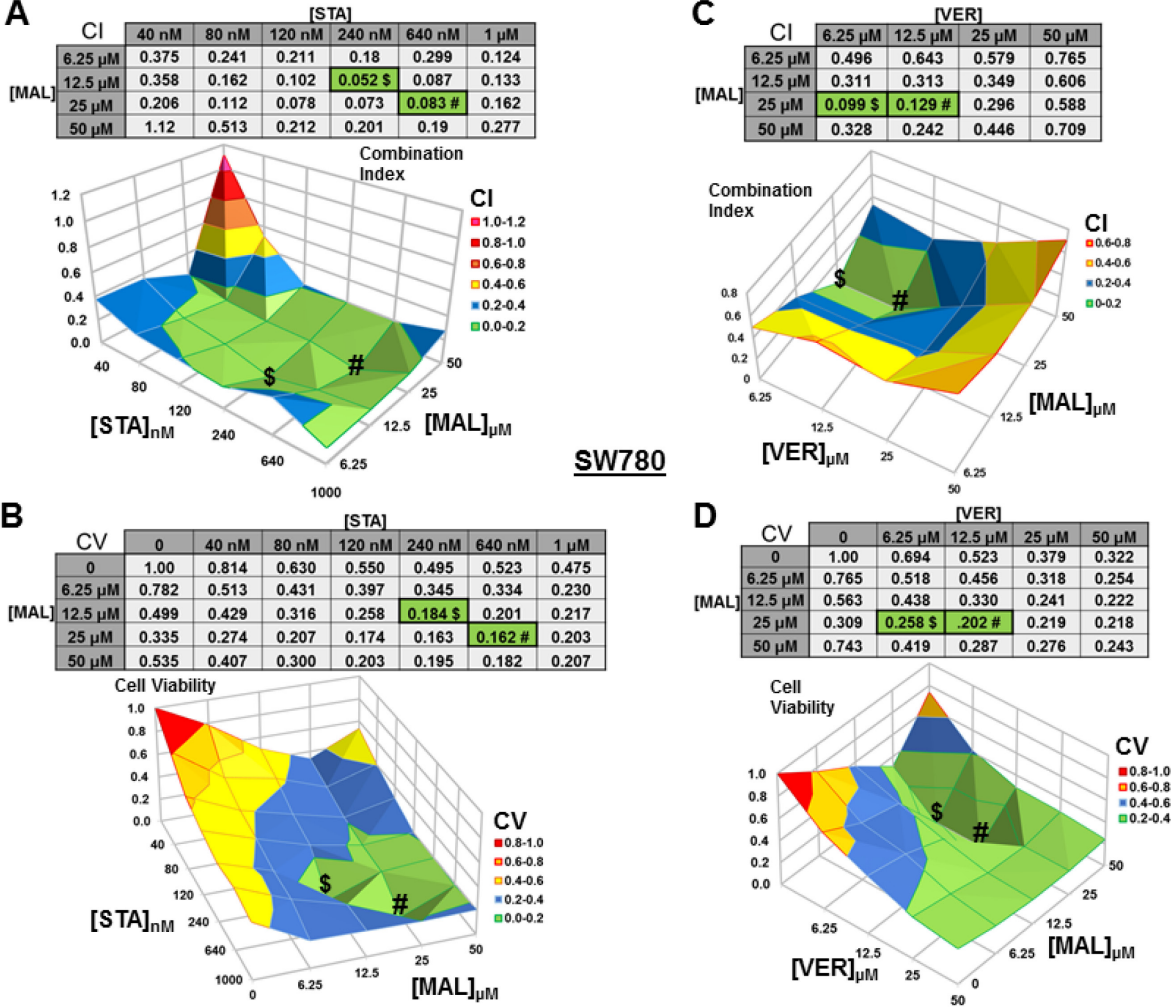
Combinations of HSP70 and HSP90 inhibitors synergistically reduce SW780 cell viability SW780 cells were treated for 48 hours with a range of STA+MAL or VER+MAL concentration combinations. (**A**) STA+MAL surface graph and CI-values, (**B**) STA+MAL surface graph and CV-values, (**C**) VER+MAL surface graph and CI-values and (**D**) VER+MAL surface graph and CV-values. ^$^ and ^#^ indicate the lowest CI-value and CV-value for each treatment for each treatment, respectively, each value in the table is colored according to its position on the surface graph.

**Figure 6:**
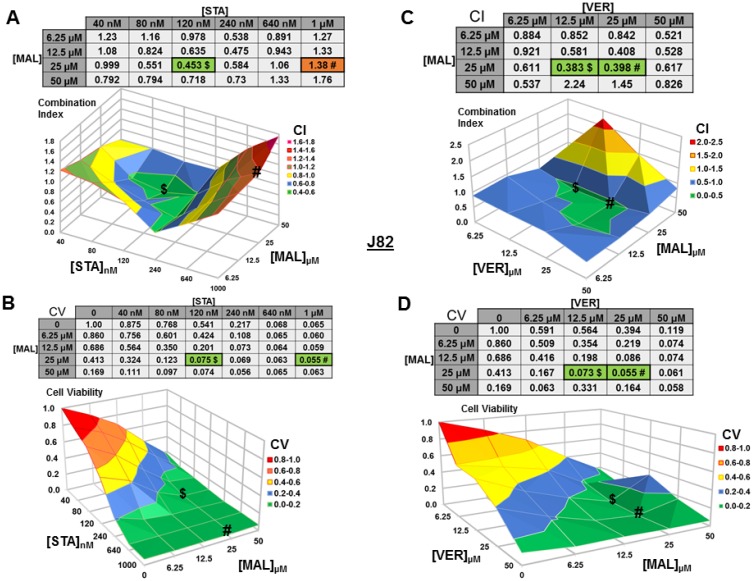
Combinations of HSP70 and HSP90 inhibitors synergistically reduce J82 cell viability J82 cells were treated for 48 hours with a range of STA+MAL or VER+MAL concentration combinations. (**A**) STA+MAL surface graph and CI-values, (**B**) STA+MAL surface graph and CV-values, (**C**) VER+MAL surface graph and CI-values and (**D**) VER+MAL surface graph and CV-values. ^$^ and ^#^ indicate the lowest CI-value and CV-value for each treatment for each treatment, respectively, each value in the table is colored according to its position on the surface graph.

We next wanted to test if combinations of STA+MAL and VER+MAL were synergistic at killing bladder cancer cells across different concentrations. Previous work in this lab showed that STA+VER were indeed synergistic at reducing cell viability [34]. To carry this out, we plated out each cell line in 96-well plates and treated them with different drug concentration combinations of STA and MAL or VER and MAL for 48 hours then harvested. Plates were then assayed for cell viability via resazurin salt reduction and crystal violet incorporation methods as previously described. Data from the crystal violet assays are shown (Figures 3–6). The combination index (CI) which signifies synergy (CI < 1) or antagonism (CI > 1) for each drug combination was calculated using the CompuSyn program by Chou and Talalay [37]. For each cell line and combination, CI values and corresponding surface graphs along with cell viability (CV) values relative to DMSO control with corresponding surface graph are presented in Figures 3–6. The drug concentration combinations that produced the lowest CI ($) values and CV (#) values are labeled on each surface graph.

For UMUC3 cells treated with STA and MAL, all concentration combinations gave CI < 1, indicating synergy (Figure 3A). The combination of 40 nM STA and 25 μM MAL was the most synergistic (CI = 0.052), however the combination that reduced cell viability the most was 240 nM STA and 25 μM MAL (CV = 0.074; Figure 3B). For VER and MAL treated UMUC3 cells, almost all concentration combinations showed synergy with the most synergistic combination of 25 μM VER and 25 μM MAL (CI = 0.107) also reducing cell viability to the lowest level (CV = 0.114; Figure 3C, 3D). In both UMUC3 experimental sets, 50 μM MAL alone did not significantly reduce cell viability similar to 10 μM MAL for 72 hours in Figure 1A, suggesting MAL may have a protective effect at higher concentrations in these cells. This produced a valley feature on both cell viability surface maps. It also should be noted that 50 μM MAL was at its limit of solubility as it appeared quite cloudy when diluted into DMEM.

For T24 cells treated with STA and MAL, synergy was observed for a group of concentration combinations with the most synergistic combination of 40 nM STA and 12.5 μM MAL (CI = 0.07) found in a crevice on the surface graph (Figure 4A). The combination of 40 nM STA and 50 μM MAL (CV = 0.149) was the most cytotoxic (Figure 4B). For VER and MAL treated T24 cells, several concentration combinations showed synergy with combinations of 6.25 μM and 12.5 μM VER with 25 μM MAL being the most synergistic (CI = 0.43) and cytotoxic (CV = 0.057, 0.047; Figure 4C, 4D).

For SW780 cells treated with STA+MAL, almost all concentration combinations showed synergy with 240 nM STA and 12.5 μM MAL being the most synergistic (CI = 0.052; Figure 5A). The combination of 640 nM STA and 25 μM MAL was the most cytotoxic (CV = 0.162). This is the only instance that we did not observe where the lowest CI and CV shared an STA or MAL concentration and directly aligned on the surface graphs (Figure 5B). For VER+MAL treated cells, all concentrations combinations showed synergy with 6.25 μM VER and 25 μM MAL being the most synergistic (CI = 0.099). The combination of 12.5 μM VER and 25 μM MAL reduced cell viability the most. Both the CI and CV shared 25 μM MAL while the VER concentration varied causing the values to align on the surface map (Figure 5C, 5D).

For J82 cells treated with STA+MAL, several synergistic concentration combinations were observed with 120 nM STA and 25 μM MAL showing the most synergy (CV = 0.453; Figure 6A). Curiously, the most cytotoxic combination, 1 μM STA and 25 μM MAL (CV = 0.056), was not calculated to be synergistic (CI = 1.38), likely related to the reduction in cell viability observed in the surrounding concentration combinations (Figure 6B). For VER+MAL treated cells, all but two concentration combinations showed synergy with 12.5 μM VER and 25 μM MAL being the most synergistic (Figure 6C). The most cytotoxic concentration combination, 25 μM VER and 25 μM MAL (CV = 0.055; Figure 6D), was located next to the lowest CI on the surface graph.

These results show that simultaneously targeting HSP90 and HSP70 or dual targeting of HSP70 can be synergistic across a range of concentration combinations. However, to achieve the greatest reduction in cell viability in each cell line the total concentrations of STA+MAL exceeded 25 μM, while total concentrations of VER+MAL were between 31.25 μM and 50 μM. Cell viability line graphs with standard error bars for each cell line are given in Supplementary Figures 5–8.

Additionally, to confirm the effectiveness of the concentration combinations that produced the lowest CI and CV values in UMUC3 and T24 cells we assayed cell viability over a 72-hour time course (Supplementary Figures 9 and 10). For UMUC3 cells each concentration combination was effective at reducing cell viability and not allowing the development of resistance suggesting cytotoxicity. The combination of 40 nM STA and 25 μM MAL was the most effective along with 240 nM STA by itself. For T24 cells each concentration combination was effective at reducing cell viability, however signs of cytostaticity were present as 72-hour levels were not significantly less than 24-hour levels. The most effective treatment was shown to be 25 μM MAL by itself.

### Targeting HSP70 and HSP90 alter protein levels of key cellular components in MIBC cells

**Figure 7:**
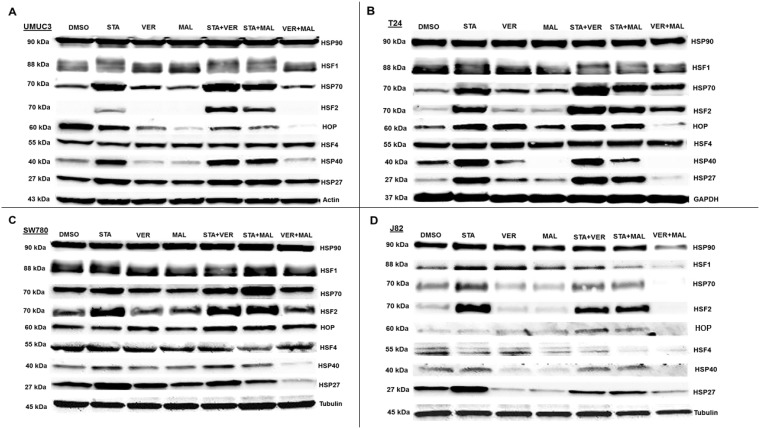
HSP70 and HSP90 inhibitors alter HSP levels in MIBC cell lines (**A**) UMUC3, (**B**) T24, (**C**) SW780 and (**D**) J82 cells were treated with either 1 μM STA, 10 μM VER, 10 μM MAL, 1 + 10 μM STA+VER, 1 + 10 μM STA+MAL or 10 + 10 μM VER+MAL for 24 hours. Cells were lysed, followed by protein quantification and western-blot analysis.

To gain insight into the molecular mechanisms of each drug and combination, we looked for changes in protein levels of key cellular components after 24 hours of treatment. The first set of proteins analyzed were comprised of HSPs and the transcription factors (HSFs) that regulate expression of HSPs (Figure 7). Inhibition of HSP90 by STA induced the expression of complementary HSPs, such as HSP70, HSP40 and HSP27, in most samples where STA was given alone or in combination with VER or MAL. The combination of VER+MAL only increased the expression of pan HSP70 in T24 cells, however for the most part; VER+MAL did not increase the levels of pan HSP70, HSP40 and HSP27 in the other cell lines. In fact, VER+MAL decreased the levels of pan HSP70 and HSP27 in J82 cells, HSP40 in T24 and SW780 cells, and HSP27 in SW780 cells. Strangely, pan HSP90 levels appeared to remain fairly constant across all conditions except in VER+MAL treated J82 cells. HOP, a co-chaperone that organizes HSP70 and HSP90 interactions, showed a varied pattern across each cell line with VER+MAL reducing HOP levels in UMUC3, T24, and J82 cells. The master transcription factor, HSF1 displayed an upward mobility shift, an indicator of activation, in STA treated UMUC3 and T24 cells. This HSF1 shift, however, was not readily observed in SW780 or J82 cells. Combining STA with either VER or MAL did not repress the HSF1 mobility shift. Conversely, VER+MAL did not induce an HSF1 mobility shift in any of the cell lines and actually reduced HSF1 levels in J82 cells. Levels of HSF4, a negative regulator of HSF1 [38], remained fairly constant across all conditions, except in J82 cells where scarce levels were further depleted by all drug treatments except VER. More remarkable, levels of the stress transcription factor, HSF2, were greatly increased in each cell line treated with STA and when combined with either VER or MAL. This effect was not seen in VER or MAL treated cells, although VER+MAL treated T24 cells did show increased HSF2 levels. To our knowledge this HSF2 induction phenomenon by inhibitors targeting HSP90 or HSP70 has not been previously reported. This observation may indicate a mechanism of how cancer cells increase their levels of HSPs in response to reduced chaperone activity.

**Figure 8:**
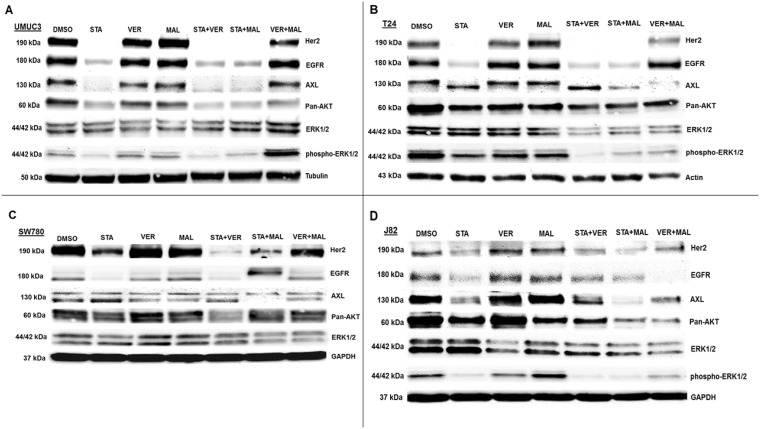
HSP70 and HSP90 inhibitors alter kinase protein levels in MIBC cell lines (**A**) UMUC3, (**B**) T24, (**C**) SW780 and (**D**) J82 cells were treated with either 1 μM STA, 10 μM VER, 10 μM MAL, 1 + 10 μM STA+VER, 1 + 10 μM STA+MAL or 10 + 10 μM VER+MAL for 24 hours. Cells were lysed, followed by protein quantification and western-blot analysis.

The second set of proteins analyzed focused on growth promoting kinases (Figure 8). Here we observed that VER+MAL reduced the levels of EGFR in SW780 and J82 cells, AXL in T24 and SW780 cells pan-AKT in UMUC3, T24 and J82 cells, and ERK1/2 in T24, SW780 and J82 cells. STA reduced the levels of Her2 and EGFR in all 4 cell lines, AXL in UMUC3 and J82 cells, and pan-AKT in UMUC3 and J82 cells. In STA treated T24 cells AXL showed a downward mobility shift suggesting a dephosphorylated state. STA+MAL also reduced overall AXL levels. STA did not alter ERK1/2 levels in any of the cell lines consistent with ERK1/2 not being an HSP90 client kinase [15, 39]. However, phosho-ERK1/2 levels in UMUC3 cells were reduced by STA, VER and MAL but not increased by VER+MAL. Moreover, phosho-ERK1/2 levels in T24 and J82 cells were reduced by both STA and VER+MAL together suggesting that several growth-promoting kinases depend on both HSP70 and HSP90 chaperoning to prevent degradation and loss of upstream kinase activity.

**Figure 9:**
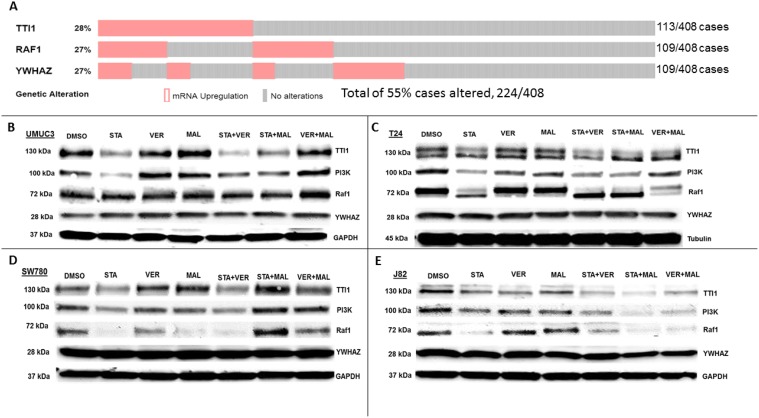
HSP70 and HSP90 inhibitors alter protein levels of bladder cancer overexpressed HSP90-interactors in MIBC cell lines (**A**) The 3 most overexpressed HSP90-interactor genes in The Cancer Genome Atlas provisional bladder cancer dataset. (**B**) UMUC3, (**C**) T24, (**D**) SW780 and (**E**) J82 cells were treated with either 1 μM STA, 10 μM VER, 10 μM MAL, 1 + 10 μM STA+VER, 1 + 10 μM STA+MAL or 10 + 10 μM VER+MAL for 24 hours. Cells were lysed, followed by protein quantification and western-blot analysis.

HSP70 and HSP90 interact with many of the same proteins and since the HSP90 interactome is well curated and defined [40], the third set of proteins that we analyzed focused on the 3 most overexpressed HSP90 interacting protein-encoding genes in The Cancer Genome Atlas provisional bladder cancer dataset [41]. TTI1, RAF1 and YWHAZ combined are found to be overexpressed in 55% of all bladder cancer cases (Figure 9A). TTI1, an HSP90 co-chaperone that helps fold phosphoinositide 3’-kinase-related kinases (PIKKs) [42], was found to be overexpressed in 28% of bladder cancer cases. STA reduced TTI1 levels in all 4 cell lines, while VER+MAL also reduced it in T24 and J82 cells (Figure 9B–9E). VER and MAL alone reduced TTI1 in J82 cells and further reduced it when combined with STA, suggesting a functional relationship with HSP70. These observations are notable since HSP90 co-chaperones levels typically are not reduced by inhibiting HSP90 (Figure 7). To further explore this observation, we blotted for PI3K, a PIKK family member that drives cell growth, and saw a similar pattern to TTI1 in each cell line suggesting that PI3K and TTI1 levels indeed coincide. Curiously, TTI1 was observed as a single band in UMUC3 and SW780 cells and as a doublet in T24 and J82cells. RAF1, a pro-growth protein kinase and HSP90 client, is overexpressed in 27% of bladder cancer cases. STA reduced levels of RAF1 in T24, SW780 and J82 cells. In T24 cells this reduction was also noted by an increased mobility shift downwards suggesting a dephosphorylated state. In SW780 cells, MAL alone decreased RAF1 levels, yet somehow STA+MAL increased RAF1 levels. VER+MAL reduced RAF1 levels in T24 and J82 cells, again suggesting a dependency on HSP70. YWHAZ, a phospho-peptide binding-protein within the 14-3-3 structural family, binds HSP90 with an unknown function and is overexpressed in 27% of cases [43, 44]. Remarkably however, YWHAZ levels were not affected by any drug treatment in all 4 cell lines suggesting it may be an interactor that does not require HSP90 or HSP70 to maintain its levels in the cell. The reduction in TTI1 and PI3K levels by STA to our knowledge has not been previously reported and suggests another mechanism by which inhibition of chaperone activity depletes pro-growth signaling components.

Also, to test the effects of concentrations combinations that showed the greatest synergy and reduction in cell viability in UMUC3 cells we blotted for a representative set of proteins (Supplementary Figure 11). Here we observed that 40 and 240 nM STA combined with 25 μM MAL reduced levels of Her2, TTI1 and RAF1 but increased levels of HSP70. The combination of 25 μM VER and 25 μM MAL did not noticeably alter the levels of any protein blotted for as compared to DMSO control. We also probed for both pro- and anti-apoptotic markers (Supplementary Figures 12 and 13).

Besides further showing that each cell line responds differently to HSP90 and HSP70 inhibitors, our western blotting results provide insight into how dual targeting of HSP70 may reduce cell viability. Like STA, VER+MAL reduced the levels of several growth promoting kinases. Unlike STA, though, VER+MAL did not typically increase the levels of HSPs but on occasion decreased them. Thus, the strategy of targeting HSP70 may be useful in preventing the development of drug resistance.

### Targeting HP70 and HSP90 alter the transcriptional heat shock response

**Figure 10:**
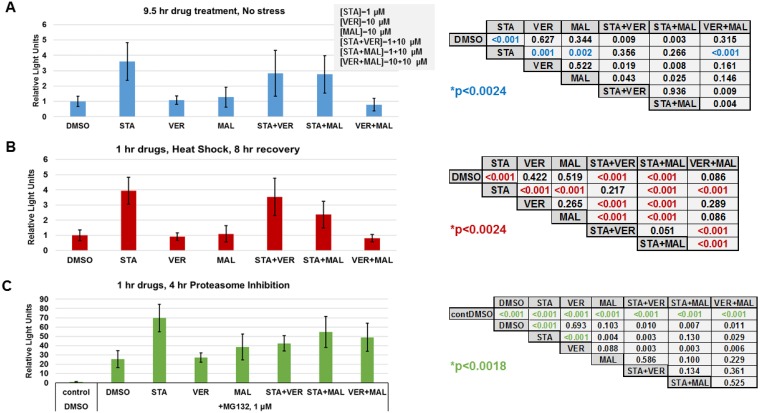
HSP70 inhibitors do not increase the heat shock response UMUC3 cells were transfected with a reporter plasmid encoding NanoLuc luciferase driven by a *HSP70B’* promoter. (**A**) Cells were treated with the indicated drug concentrations for 9.5 hours then harvested and assayed for NanoLuc activity. Each treatment was compared using a *T*-test with a Bonnferroni correction for multiple testing (21 comparisons) *p* ≤ 0.0024. (**B**) Cells were treated with the indicated drugs for 1 hour, heat shocked for 30 minutes at 45^°^ C and allowed to recover for 8 hours before being harvested. Each treatment was compared using a *T*-test with a Bonnferroni correction for multiple testing (21 comparisons) *p* ≤ 0.0024. (**C**) Cells were treated with the indicated drugs for 1 hour, then given 1 μM MG132 for 4 hours and harvested. Each treatment was compared using a *T*-test with a Bonnferroni correction for multiple testing (27 comparisons) *p* ≤ 0.0018. A comparison of control, non-stressed cells (A), and heat shocked cells (B) is shown in Supplementary Figure 14.

To further determine if targeting HSP70 does not induce the heat shock response, the last set of experiments we carried out focused on determining how each drug and combination affected the cellular heat shock response at the level of gene transcription. Typically, inhibitors that target the N-terminal of HSP90, such as STA, activate the heat shock response and the expression of fellow HSPs [20]. Both heat shock and proteasome inhibition are strong inducers of the stress response [45]. To determine the effects of each drug and combination in heat shocked or proteasome inhibited cells, we utilized a reporter plasmid encoding destabilized NanoLuc luciferase driven by an HSP70B’ promoter. UMUC3 cells were transfected overnight, treated with drugs for 1 hour and then either left alone, exposed to heat shock followed by an 8-hour recovery or incubated with proteasome inhibitor MG132 for 4 hours as described in materials and methods. Control cells that were left alone were harvested 9 hours after drug treatment. Data from drug treated controls showed that STA induced detectable expression of the heat shock reporter above DMSO, VER, MAL and VER+MAL treated cells (Figure 10A). In heat-shocked cells, STA, STA+VER and STA+MAL amplified the expression of the heat shock reporter above VER, MAL and VER+MAL (Figure 10B). Proteasome inhibition in MG132 treated cells strongly induced expression of the heat shock reporter (Figure 10C). STA amplified heat shock response the most across all treatments. These results showed that STA induces a low-level stress response in cancer cells. Moreover, STA amplifies the stress response when cells are exposed to

heat shock or proteasome inhibition. VER and MAL did not induce or amplify the stress response confirming that targeting HSP70 does not increase the stress response. However, when combined with STA, VER and MAL did not prevent amplification of the response. VER+MAL did not increase reporter activity in response to heat shock.

## DISCUSSION

In this report we explore the use of inhibitor combinations targeting both HSP70 and HSP90 to treat MIBC. Dual targeting of HSP70 by VER+MAL proved effective at reducing cell viability and depleting the levels of some growth promoting kinases. Furthermore, VER+MAL did not induce the expression HSPs or amplify the cytoprotective stress shock response, in contrast to HSP90 inhibition by STA. Despite requiring elevated concentrations of VER+MAL to be effective, our work demonstrates that targeting HSP70 chaperone activity may be an effective strategy for combatting MIBC. Nonetheless improved versions of these inhibitors must be developed to improve solubility, reduce therapeutic concentrations and increase specificity for them to be clinically relevant.

In our synergy experiments, we observed that the lowest CI and CV values typically shared at least one drug concentration causing them to directly align with each other across the surface graph. This alignment suggests that the most effective concentration combinations exist within a “valley of cytotoxicity”. The implications of this observation in the cancer clinic would be the necessity to optimize drug-dosing schedules to maintain the primary drug at a steady concentration while levels of the secondary drug could vary along a range of concentrations. This understanding could help in designing clinical trials that must consider the pharmacodynamics and pharmacokinetics of each drug within a combination.

Despite STA+MAL and VER+MAL demonstrating synergy across a number of combination concentrations, increasing the effective cytotoxicity per unit of total drug is still needed. Current efforts to develop inhibitors that trap either HSP70 or HSP90 in “closed” conformations that block both active (ATP-fueled) and passive-holdase (ATP-independent) chaperone functions and thereby further reduce protein-folding capacity could help address this need [46, 47]. Additionally, inhibiting HSP70 by trapping it in a “closed” conformation may allow for the targeting of HSF1, a robust enabler of malignancy. As HSP70 directly represses HSF1 through binding its transcription activation domain [48], trapping HSP70 on HSF1 could prevent increased expression of HSPs in proliferating cancer cells.

Despite not confirming nuclear accumulation and DNA binding, our finding that STA increased the expression of HSF2 was unexpected and may be another mechanism that allows MIBC cells to resist STA treatment. Although not able to initiate the stress response on its own, HSF2 synergistically enhances gene expression by complexing with HSF1 on HSP gene promoters [49]. This suggests that elevated levels of cytoprotective HSPs seen in STA treated cells may be due in part to increased expression of HSF2 (Figure 7). Thus, resistance to Hsp90 N-terminal inhibitors may involve more than just unattenuated HSF1 activity [20, 50]. Further studies, however, are required to determine if this HSF2 induction phenomenon occurs in other cancer cell lines and if it can be prevented.

Finally, our observations that TTI1 and PIK3Ca levels were reduced by STA and to a lesser extent by VER and MAL suggests that both proteins require chaperone activity similar to clients. Yet, TTI1 is understood to be a co-chaperone that helps Hsp90 fold and assemble PIKKs, such as PIK3Ca, into large protein-complexes that signal for growth and survival [42]. Thus, implying that the loss of TTI1 results in loss of PIK3Ca. This observation however does not exactly fit with the findings of Takai *et al*. that showed that TTI1 levels were not affected by the first generation Hsp90 inhibitor, geldanamycin, in HeLa S3 cells suggesting that our findings may be MIBC cell line specific [42]. Another possible mechanism is that prolonged exposure to STA reduces growth factor signaling allowing for degradation of TTI1 by Fbxo9 [51], a ubiquitin-ligase that interacts with HSP90 [15]. No matter the mechanism though, the fact that TTI1 (28%) is overexpressed and kinase-PIK3Ca signaling (72%) is altered in a significant number of bladder cancer cases indicates that reducing the chaperone activity that supports this axis may be viable approach to treating MIBC [7].

In summary, targeting HSP90 is a worthwhile approach to disrupting multiple oncogenic signaling pathways that drive cancer, however particular attention must be given to preventing upregulation of the compensatory heat shock response that may further enable oncogenesis [52]. Dual targeting of HSP70 highlighted here may be an approach to reduce oncogenic signaling and not induce the heat shock response. Despite the drug concentrations of VER and MAL employed in this study being above clinically relevant levels, this study provides a mechanistic framework to more effectively target HSP70 with small molecule inhibitors going forward.

## MATERIALS AND METHODS

### Cell culture

Bladder cancer cell lines were recently purchased from ATCC: UMUC3 (# CRL-1749), T24 (# HTB-4), SW780 (# CRL-2169) and J82 (# HTB-1). Cells were cultured in MEM (Gibco) supplemented with 10% FBS (HyClone^™^) and Penn/Strep (Sigma) antibiotic solution under 37° C, 5% CO_2_ conditions. When confluency was reached, cells were passaged using TrypLE Express (Gibco) trypsinization solution.

### Cell viability

Cells were aliquoted at approximately 10,000 cells/well in a final volume of 100 μl MEM in 96-well plates. Twenty-four hours after plating, plates were treated with either STA9090 (Synta Pharmaceuticals), VER155008 (Sellek Chemicals), MAL3-101 (AChemtek), or a combination and incubated for 24, 48, or 72hrs with drug(s). Each drug treatment was repeated (*n* = 8) per plate. At each time point, 20 μl of resazurin salt (Sigma) at 0.15mg/mL was added to each well and incubated for 1 hour. Plates were then read on a SpectraMax 220 spectrometer at 560–590 nm fluorescence to determine cell viability based on aerobic respiration [35]. After reading the resazurin results, each plate was washed 3X with PBS, then 50 μl of 0.2% crystal violet (Gold Biotechnology) in 2% ethanol was added to each well and incubated for 15 minutes at room temperature. Plates were washed 3X with ddH_2_O followed by addition of 100 μl of 1% SDS solution. Plates were gently rocked for 4 hours at room temperature, then read for absorbance at 570 nm to determine viability based on protein and DNA content [53, 36]. For both cell viability assays, values were normalized to the DMSO treatment at 24 hours. This was done by dividing all values by the average of the DMSO wells on the 24-hour plate. Each drug treatment condition was then averaged, and standard deviation calculated. Each treatment was compared using a *T*-test with a Bonnferroni correction for multiple testing. All calculations were performed using Excel (Microsoft).

### Drug synergy

Cells were seeded into 96-well plates at approximately 10,000 cells/well and allowed to grow for 24 hours. Drug treatment combinations were added to each well with repeats (*n* = 6). Plates were processed for cell viability 48 hours after treatment using both resazurin and crystal violet assays. Cell viability results were normalized to DMSO controls and then inputted into the CompuSyn program that calculated each Combination Index (CI) value using the Chou and Talalay method [37], where CI < 1 indicates synergy, CI = 1 indicates additive effect and CI > 1 indicates antagonism.

### Western blots

To determine protein expression, culture dishes were removed of media and cells were lysed using buffer solution consisting of 50 mM Tris pH 7.6, 50 mM NaCl, 2 mM EDTA, 0.5% Tritonx100, 0.1% SDS, 0.05% Deoxycholate, and 2 mM Phenylmethanesulfonyl fluoride with additional Halt Protease and Phosphatase Inhibitor (Thermo Scientific). The resulting lysates were spun at maximum speed (13,000 rpm) in a benchtop centrifuge for 15 minutes at 4° C. Supernatants were collected and analyzed for protein concentration using Pierce BCA Protein Assay Kit (Thermo Scientific). Equal amounts of lysates (30–40 μg, depending on cell line) were loaded at on 4–20% Criterion TGX Stain-Free Precast Gels (Bio-Rad) and ran at 100V, followed by transfer to a nitrocellulose membrane using the Trans-Blot Turbo RTA Transfer Kit and transfer system (Bio-Rad). Membranes were rinsed with ddH_2_0 and blocked with 5% non-fat powdered milk (Boston Bioproducts). Primary antibodies at 1:1,000 dilution were added and incubated overnight at 4° C with gentle rocking. Next day membranes were washed 3X with TBSt (10 mM Tris-HCl pH 7.6, 150 mM NaCl, and 0.1% Tween-20) with 0.25% milk, then incubated with 1: 2,000 secondary-HRP antibody for 2 hours. Membranes were washed 3X with TBST, developed with ECL western blotting solution (Pierce) and imaged on a UVP ChemiDoc-lt Imaging System. ImageJ software was used to process all western blot images. Primary antibodies were purchased from Santa Cruz Biotechnology: Axl (C-20), Her2 (3B5), HSF1 (E-4), HSF2 (G-11), HSF4 (N-12), HSP27 (G3.1), HSP40 (B-3), HOP (28), HSP70 (F-3), HSP90 (H-114), RAF1 (E-10), and YWHAZ (1B3), GAPDH (A-3), Actin (C4), and Tubulin (C-20); Cell Signaling Technologies ERK1/2, phosph-ERK1/2 (T202/Y204), AKT and PI3K; Sigma Aldrich: TTI1 (SAB4301632). Secondary anti-mouse and anti-rabbit antibodies were purchased from Boston BioProducts.

### The Cancer Genome Atlas

Hsp90 interactor gene expression levels were determined by mining the cBioPortal for Cancer Genomics site that contains The Cancer Genome Atlas datasets [41, 54]. The Bladder Urothelial Carcinoma (TCGA, provisional) dataset was selected along with mRNA Expression z-Scores toggle. To identify the cases where TTI1, RAF1 and YWHAZ mRNA expression was 2 standard deviations above the mean, the scripts TTI1:EXP>2, RAF1:EXP>2 and YWHAZ: EXP>2 were entered.

### Heat shock response reporter

Cells were seeded in 96-well plates at approximately 10,000 cells/well and allowed to grow for 18 hours. Cells were transfected for 16 hours with a stress response plasmid containing an Hsp70b promoter driving expression of destabilized a NanoLuc (pGL4-hsp70b-NL-2CP/ARE) construct along with a transfection control plasmid (pCMV-βGal). Drugs were aliquoted into each well with each drug condition repeated (*n* = 8) for 1 hour. For heat shock, cells were placed in an incubator at 45° C for 30 minutes then allowed to recover for 8 hours at 37° C before being harvested. For proteasome inhibition, 1 μM MG132 was added then incubated at 37° C for 4 hours before being harvested. Cells were harvest by removing media, adding 100 μL of reporter lysis buffer (25 mM Bicine, pH 7.6, 0.20% Triton X-100, 0.05% Tween 20) and placing in –80° C freezer. To determine reporter activity, plates were thawed and 50 μL of lysate from each well was aliquoted into a corresponding well on a clean white 96-well plate. Nano-Glo luciferase reagent (10 μL) was added to each well on the white 96-well plate, then incubated at room temperature for 5 minutes before being read on a SpectraMax 220 luminometer. To determine transfection efficiency, 40 uL of β-gal assay reagent (200 mM NaPO_4_, 2 mM MgCl_2_, 100 mM β-mercapethanol, 1.33 mg/mL ONPG) was added to each well in the original cell culture plate, incubated at 37° C for 20 minutes and then read for absorbance at 420 nm. Relative reporter activity was calculated by dividing the luciferase readout value by the β-gal assay readout. For heat shock experiments, activity was normalized by dividing all values by the average of the DMSO control. For proteasome inhibition experiments, activity was normalized by dividing all values by the average of the control DMSO (no MG132). Each condition was averaged, and the standard deviation calculated. Each treatment was compared using a *T*-test with a Bonnferroni correction for multiple testing. All calculations were performed using Excel (Microsoft).

## SUPPLEMENTARY MATERIALS FIGURES AND TABLES



## Abbreviations

MIBC: Muscle invasive bladder cancer
HSPs: heat shock proteins
HSP70: 70 kDa heat shock protein
HSP90: 90-kDa heat shock protein
VER: VER155008
MAL: MAL3-101
STA: STA-9090
CI: Combination Index
CV: cell viability
PIKKs: phosphoinositide 3'-kinase-related kinases
HSFs: heat shock factors.
