# Supplementary material for: Dual targeting of HSP70 does not induce the heat shock response and synergistically reduces cell viability in muscle invasive bladder cancer
Source: Oncotarget. 2018 Aug 24;9(66):32702–17. doi: 10.18632/oncotarget.26021 (PMC6135696; doi:10.18632/oncotarget.26021)
Supplement: Supplementary file 1 [file oncotarget-09-32702-s001.pdf]

Dual targeting of HSP70 does not induce the heat shock response and synergistically reduces cell viability in muscle invasive bladder cancer

SUPPLEMENTARY MATERIALS

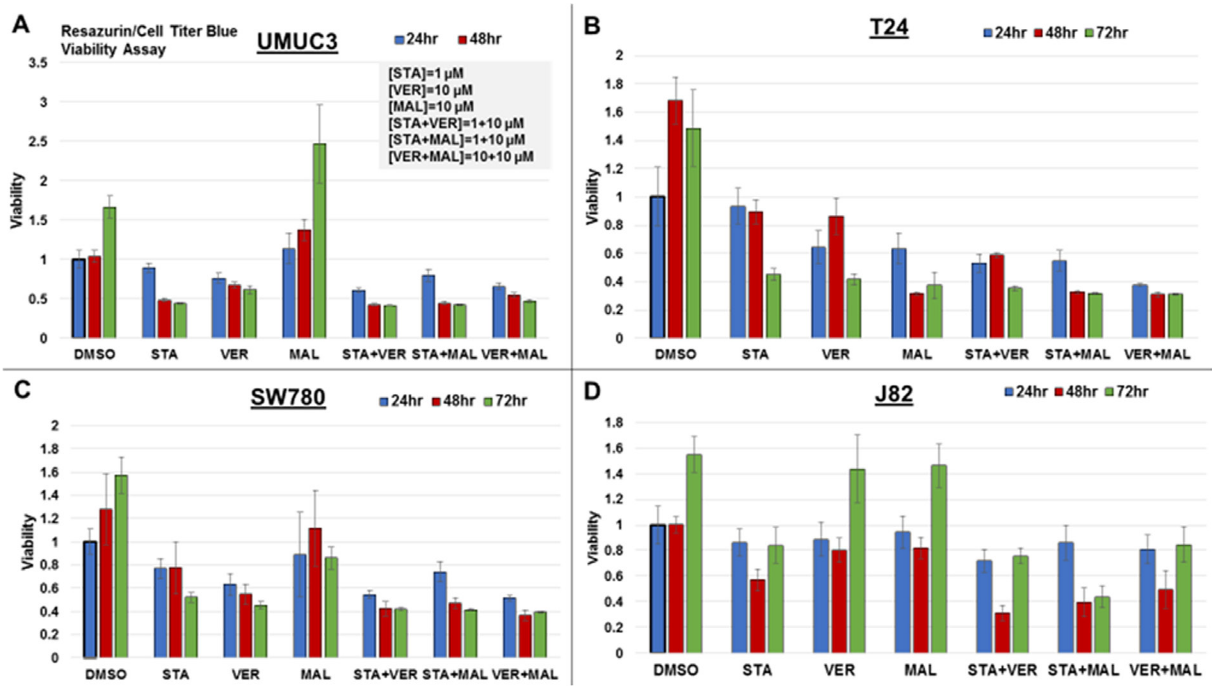

Supplementary Figure 1: Combinations of Hsp70 and Hsp90 inhibitors reduce cell viability to different degrees over time. MIBC cell lines (**A**) UMUC3, (**B**) T24, (**C**) SW780 and (**D**) J82 were plated and treated with STA, VER and MAL alone or in combination for 24, 48, and 72 hours. Cell viability was determined by resazurin and normalized to the 24-hour DMSO control for each cell line ± standard deviation.

UMUC3	24hr-48h	48h-72h	24h-72h
STA	0.077	0.053	0.191
VER	0.117	<.001	<.001
MAL	0.039	<.001	<.001
STA+VER	0.002	0.002	0.180
STA+MAL	0.030	<.001	0.007
VER+MAL	0.027	<.001	0.011

T24	24hr-48h	48h-72h	24h-72h
STA	<.001	0.013	0.004
VER	<.001	<.001	0.484
MAL	0.021	0.013	0.538
STA+VER	0.484	<0.001	0.008
STA+MAL	0.026	0.110	0.003
VER+MAL	0.008	0.074	<.001

p<0.017

SW780	24hr-48h	48h-72h	24h-72h
STA	0.137	0.626	0.017
VER	0.044	0.451	0.013
MAL	0.106	0.200	<.001
STA+VER	<.001	<.001	<.001
STA+MAL	0.091	0.028	0.001
VER+MAL	0.218	0.077	0.001

J82	24hr-48h	48h-72h	24h-72h
STA	<.001	0.248	0.005
VER	0.218	<.001	<.001
MAL	0.741	<.001	<.001
STA+VER	<.001	<.001	0.003
STA+MAL	<.001	0.013	<.001
VER+MAL	<.001	0.859	<.001

Supplementary Figure 2: Statistical analysis of each drug treatment across each time point for Figure 2. Student *T*-tests were used to compare each timepoint. Significance was noted as $p < 0.05/3$ comparisons = 0.017. Values are color coded based on the timepoint that had the lowest cell viability.

UMUC3

24hr	STA	VER	MAL	STA+VER	STA+MAL	VER+MAL
DMSO	0.017	0.437	0.132	0.145	0.044	0.118
STA		0.120	0.002	0.378	0.469	0.720
VER			0.068	0.511	0.312	0.343
MAL				0.022	0.005	0.023
STA+VER					0.789	0.696
STA+MAL						0.841

48hr	STA	VER	MAL	STA+VER	STA+MAL	VER+MAL
DMSO	<0.001	<0.001	0.003	<0.001	<0.001	<0.001
STA		0.017	<0.001	0.087	0.858	0.264
VER			<0.001	<0.001	0.062	0.001
MAL				<0.001	<0.001	<0.001
STA+VER					0.139	0.595
STA+MAL						0.281

72hr	STA	VER	MAL	STA+VER	STA+MAL	VER+MAL
DMSO	<0.001	<0.001	0.060	<0.001	<0.001	<0.001
STA		0.007	<0.001	0.999	0.228	0.474
VER			<0.001	0.002	0.034	0.008
MAL				<0.001	<0.001	<0.001
STA+VER					0.119	0.312
STA+MAL						0.431

T24

24hr	STA	VER	MAL	STA+VER	STA+MAL	VER+MAL
DMSO	<0.001	0.004	0.204	0.841	0.031	<0.001
STA		0.298	0.093	0.011	0.334	0.678
VER			0.337	0.056	0.869	0.508
MAL				0.380	0.468	0.149
STA+VER					0.108	0.019
STA+MAL						0.489

48hr	STA	VER	MAL	STA+VER	STA+MAL	VER+MAL
DMSO	<0.001	<0.001	<0.001	<0.001	<0.001	<0.001
STA		0.759	<0.001	0.019	<0.001	<0.001
VER			<0.001	0.002	<0.001	<0.001
MAL				<0.001	0.442	0.162
STA+VER					<0.001	<0.001
STA+MAL						0.461

72hr	STA	VER	MAL	STA+VER	STA+MAL	VER+MAL
DMSO	<0.001	<0.001	<0.001	<0.001	<0.001	<0.001
STA		0.284	0.268	0.003	<0.001	<0.001
VER			0.865	0.026	<0.001	<0.001
MAL				0.064	<0.001	<0.001
STA+VER					0.001	<0.001
STA+MAL						0.103

Supplementary Figure 3: Statistical analysis of UMUC3 and T24 cells at each timepoint for Figure 2. Student *T*-tests were used to compare each timepoint. Significance was noted as $p < 0.05/21$ comparisons = 0.024. Values are color coded based on the treatment that reduced cell viability the most.

SW780

24hr	STA	VER	MAL	STA+VER	STA+MAL	VER+MAL
DMSO	<0.001	0.290	0.004	0.565	0.046	<0.001
	STA	0.047	0.018	0.003	0.209	0.033
		VER	0.390	0.572	0.425	0.010
			MAL	0.071	0.901	<0.001
				STA+VER	0.143	<0.001
					STA+MAL	0.042

J82

24hr	STA	VER	MAL	STA+VER	STA+MAL	VER+MAL
DMSO	<0.001	0.355	0.923	0.357	0.113	0.185
	STA	<0.001	<0.001	<0.001	0.019	<0.001
		VER	0.296	0.959	0.423	0.044
			MAL	0.295	0.093	0.190
				STA+VER	0.384	0.038
					STA+MAL	0.017

48hr	STA	VER	MAL	STA+VER	STA+MAL	VER+MAL
DMSO	0.001	<0.001	0.015	<0.001	<0.001	<0.001
	STA	0.116	0.435	0.085	0.073	0.032
		VER	0.035	0.703	0.562	0.092
			MAL	0.025	0.022	0.011
				STA+VER	0.745	0.101
					STA+MAL	0.334

48hr	STA	VER	MAL	STA+VER	STA+MAL	VER+MAL
DMSO	<0.001	0.016	0.463	<0.001	<0.001	<0.001
	STA	0.001	<0.001	0.067	0.697	0.072
		VER	0.034	<0.001	0.003	0.007
			MAL	<0.001	<0.001	<0.001
				STA+VER	0.126	0.001
					STA+MAL	0.365

72hr	STA	VER	MAL	STA+VER	STA+MAL	VER+MAL
DMSO	<0.001	<0.001	<0.001	<0.001	<0.001	<0.001
	STA	0.003	<0.001	<0.001	<0.001	<0.001
		VER	<0.001	<0.001	0.003	0.001
			MAL	<0.001	<0.001	<0.001
				STA+VER	0.675	0.689
					STA+MAL	0.960

72hr	STA	VER	MAL	STA+VER	STA+MAL	VER+MAL
DMSO	<0.001	0.157	0.553	<0.001	<0.001	<0.001
	STA	<0.001	<0.001	0.002	<0.001	0.532
		VER	0.066	<0.001	<0.001	<0.001
			MAL	<0.001	<0.001	<0.001
				STA+VER	<0.001	0.007
					STA+MAL	<0.001

Supplementary Figure 4: Statistical analysis of SW780 and J82 cells at each timepoint for Figure 2. Student *T*-tests were used to compare each timepoint. Significance was noted as $p < 0.05/21$ comparisons = 0.024. Values are color coded based on the treatment that reduced cell viability the most.

UMUC3

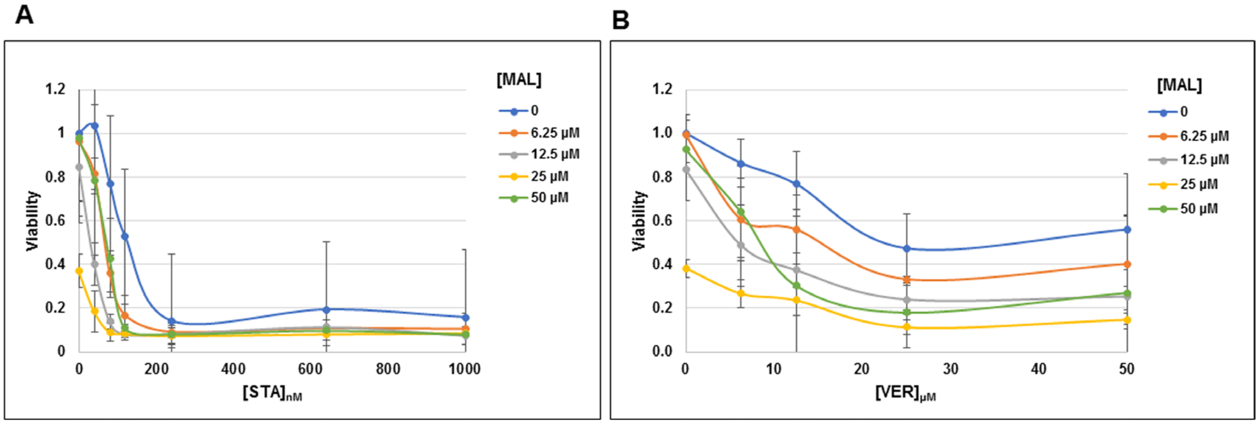

Supplementary Figure 5: HSP70 and HSP90 inhibitors synergistically reduce UMUC3 cell viability. Line graph of corresponding to Figure 3. Error bars ± standard error. (**A**) STA+MAL (**B**) VER+MAL.

T24

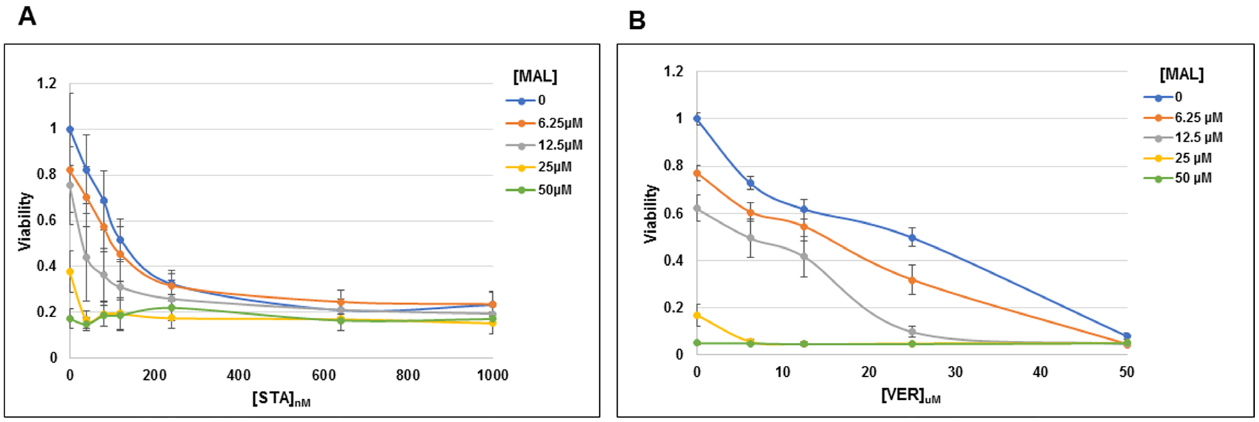

Supplementary Figure 6: HSP70 and HSP90 inhibitors synergistically reduce T24 cell viability. Line graph of corresponding to Figure 4. Error bars ± standard error. (**A**) STA+MAL (**B**) VER+MAL.

SW780

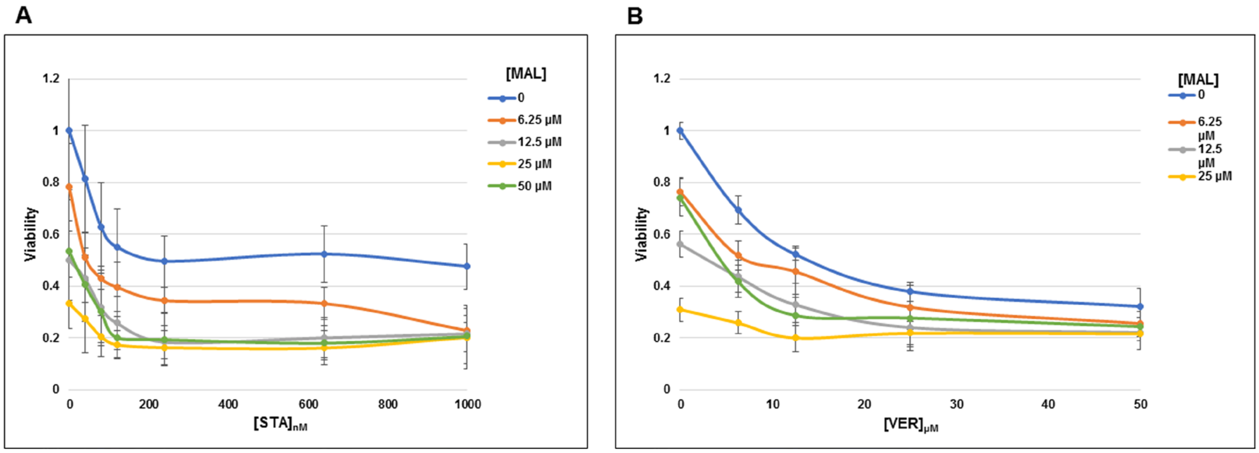

Supplementary Figure 7: HSP70 and HSP90 inhibitors synergistically reduce SW780 cell viability. Line graph of corresponding to Figure 5. Error bars ± standard error. (**A**) STA+MAL (**B**) VER+MAL.

J82

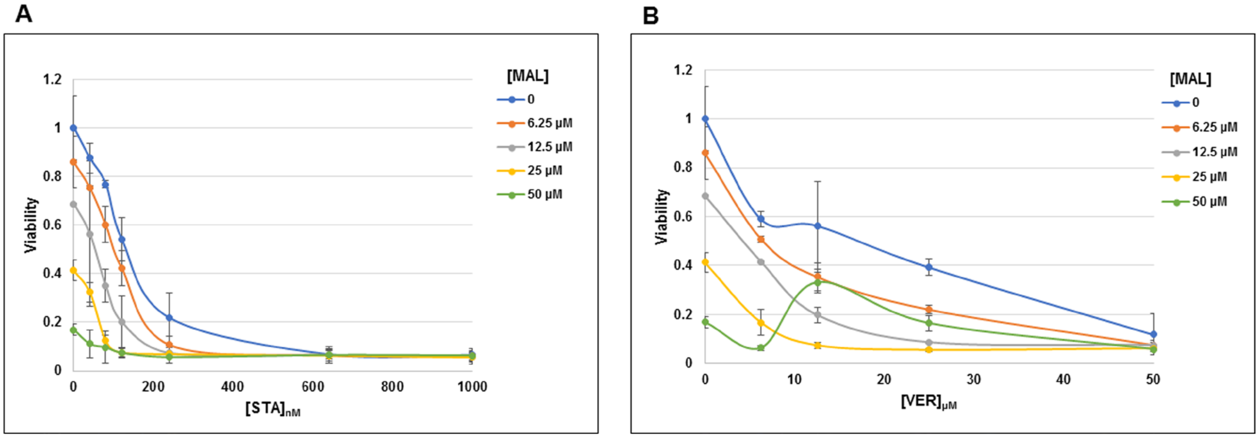

Supplementary Figure 8: HSP70 and HSP90 inhibitors synergistically reduce J82 cell viability. Line graph of corresponding to Figure 6. Error bars ± standard error. (**A**) STA+MAL (**B**) VER+MAL.

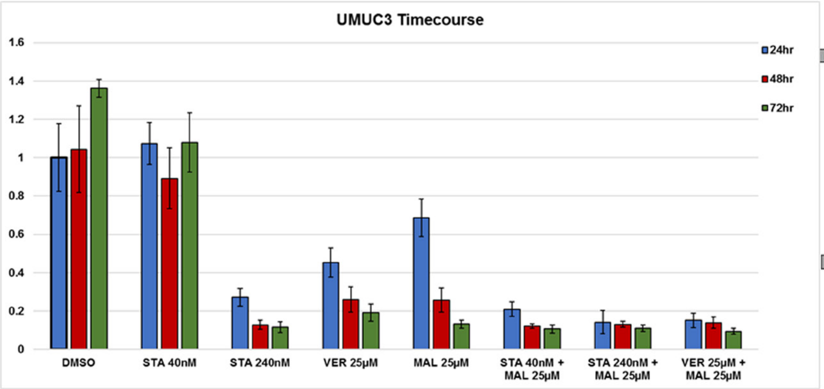

24hrs	STA 40nM	STA 240nM	VER 25uM	MAL 25uM	STA 40nM+MAL 25uM	STA 240nM+MAL 25uM	VER 25uM+MAL 25uM
DMSO	0.181	<.001	<.001	0.004	<.001	<.001	<.001
STA 40nM		<.001	<.001	<.001	<.001	<.001	<.001
STA 240nM			<.001	<.001	0.001	0.002	0.001
VER 25uM				0.002	<.001	<.001	<.001
MAL 25uM					<.001	<.001	<.001
STA 40nM+MAL 25uM						0.007	0.004
STA 240nM+MAL 25uM							0.435
VER 25uM+MAL 25uM							

*p<0.0018

48hrs	STA 40nM	STA 240nM	VER 25uM	MAL 25uM	STA 40nM+MAL 25uM	STA 240nM+MAL 25uM	VER 25uM+MAL 25uM
DMSO	0.010	<.001	<.001	<.001	<.001	<.001	<.001
STA 40nM		<.001	<.001	<.001	<.001	<.001	<.001
STA 240nM			<.001	<.001	0.217	0.360	0.155
VER 25uM				0.418	<.001	<.001	<.001
MAL 25uM					<.001	<.001	<.001
STA 40nM+MAL 25uM						0.023	0.092
STA 240nM+MAL 25uM							0.137
VER 25uM+MAL 25uM							

*p<0.0018

UMUC3	24hr-48h	48h-72h	24h-72h
STA 40nM	0.022	0.467	0.004
STA 240nM	<.001	0.251	<.001
VER 25uM	<.001	<.001	<.001
MAL 25uM	<.001	<.001	<.001
STA 40nM+MAL 25uM	<.001	0.056	<.001
STA 240nM+MAL 25uM	0.328	0.001	0.12
VER 25uM+MAL 25uM	0.266	0.002	0.005

*p<0.0023

48hrs	STA 40nM	STA 240nM	VER 25uM	MAL 25uM	STA 40nM+MAL 25uM	STA 240nM+MAL 25uM	VER 25uM+MAL 25uM
DMSO	0.010	<.001	<.001	<.001	<.001	<.001	<.001
STA 40nM		<.001	<.001	<.001	<.001	<.001	<.001
STA 240nM			<.001	<.001	0.217	0.360	0.155
VER 25uM				0.418	<.001	<.001	<.001
MAL 25uM					<.001	<.001	<.001
STA 40nM+MAL 25uM						0.023	0.092
STA 240nM+MAL 25uM							0.137
VER 25uM+MAL 25uM							

*p<0.0018

Supplementary Figure 9: Synergistic dosages of HSP70 and HSP90 inhibitors reduce UMUC3 viability over 24, 48, and 72 hours. Drug doses that gave greatest reduction in cell viability or the most synergy were given to UMUC3 cells for 24, 48, and 72-hour time points as single agents and in combination. Cell viability was determined by crystal violet and normalized to the 24-hour DMSO control for each cell line ± standard deviation. Student *t*-test values shown with a $p < 0.0018$ to determine statistical significance between drug conditions for each time point and between each time point.

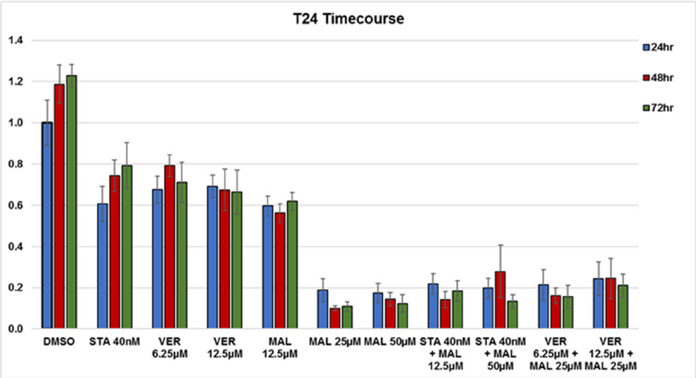

48hrs	STA 40nM	VER 6.25uM	VER 12.5uM	MAL 12.5uM	MAL 25uM	MAL 50uM	STA 40nM+MAL 12.5uM	STA 40nM+MAL 50uM	VER 6.25uM+MAL 25uM	VER 12.5uM+MAL 25uM
DMSO	<.001	<.001	<.001	<.001	<.001	<.001	<.001	<.001	<.001	<.001
STA 40nM		0.079	0.104	<.001	<.001	<.001	<.001	<.001	<.001	<.001
VER 6.25uM			0.021	<.001	<.001	<.001	<.001	<.001	<.001	<.001
VER 12.5uM				0.001	<.001	<.001	<.001	<.001	<.001	<.001
MAL 12.5uM					<.001	<.001	<.001	<.001	<.001	<.001
MAL 25uM						<.001	<.001	<.001	<.001	<.001
MAL 50uM							<.001	<.001	<.001	<.001
STA 40nM+MAL 12.5uM								0.016	0.091	0.014
STA 40nM+MAL 50uM									0.024	0.237
VER 6.25uM+MAL 25uM										0.013
VER 12.5uM+MAL 25uM										

*p<0.0010

72hrs	STA 40nM	VER 6.25uM	VER 12.5uM	MAL 12.5uM	MAL 25uM	MAL 50uM	STA 40nM+MAL 12.5uM	STA 40nM+MAL 50uM	VER 6.25uM+MAL 25uM	VER 12.5uM+MAL 25uM
DMSO	<.001	<.001	<.001	<.001	<.001	<.001	<.001	<.001	<.001	<.001
STA 40nM		0.026	0.005	<.001	<.001	<.001	<.001	<.001	<.001	<.001
VER 6.25uM			0.198	0.024	<.001	<.001	<.001	<.001	<.001	<.001
VER 12.5uM				0.072	<.001	<.001	<.001	<.001	<.001	<.001
MAL 12.5uM					<.001	<.001	<.001	<.001	<.001	<.001
MAL 25uM						0.046	<.001	<.001	<.001	<.001
MAL 50uM							<.001	<.001	<.001	<.001
STA 40nM+MAL 12.5uM								0.015	0.113	0.072
STA 40nM+MAL 50uM									0.112	<.001
VER 6.25uM+MAL 25uM										<.001
VER 12.5uM+MAL 25uM										

*p<0.0010

24hrs	STA 40nM	VER 6.25uM	VER 12.5uM	MAL 12.5uM	MAL 25uM	MAL 50uM	STA 40nM+MAL 12.5uM	STA 40nM+MAL 50uM	VER 6.25uM+MAL 25uM	VER 12.5uM+MAL 25uM
DMSO	<.001	<.001	<.001	<.001	<.001	<.001	<.001	<.001	<.001	<.001
STA 40nM		0.034	0.051	0.356	<.001	<.001	<.001	<.001	<.001	<.001
VER 6.25uM			0.337	0.015	<.001	<.001	<.001	<.001	<.001	<.001
VER 12.5uM				0.004	<.001	<.001	<.001	<.001	<.001	<.001
MAL 12.5uM					<.001	<.001	<.001	<.001	<.001	<.001
MAL 25uM						0.224	<.001	<.001	<.001	<.001
MAL 50uM							<.001	<.001	<.001	<.001
STA 40nM+MAL 12.5uM								0.048	0.339	0.043
STA 40nM+MAL 12.5uM									0.108	0.012
VER 6.25uM+MAL 25uM										0.014
VER 12.5uM+MAL 25uM										

*p<0.0010

T24	24hr-48h	48h-72h	24h-72h
STA 40nM	0.002	0.106	0.005
VER 6.25uM	0.002	0.014	0.149
VER 12.5uM	0.314	0.403	0.199
MAL 12.5uM	0.103	0.008	0.187
MAL 25uM	<.001	0.051	<.001
MAL 50uM	0.057	0.073	<.001
STA 40nM+MAL 12.5uM	<.001	0.012	0.057
STA 40nM+MAL 50uM	0.096	0.012	0.001
VER 6.25uM+MAL 25uM	0.006	0.384	0.057
VER 12.5uM+MAL 25uM	0.489	0.225	0.141

*p<0.0017

Supplementary Figure 10: Synergistic dosages of HSP70 and HSP90 inhibitors reduce T24 viability over 24, 48, and 72 hours. Drug doses that gave greatest reduction in cell viability or most synergy were given to T24 cells for 24, 48, and 72-hour time points as single agents and in combination. Cell viability was determined by crystal violet and normalized to the 24-hour DMSO control for each cell line ± standard deviation. Student *t*-test values shown with a $p < 0.0010$ to determine statistical significance between drug conditions for each time point and between each time point.

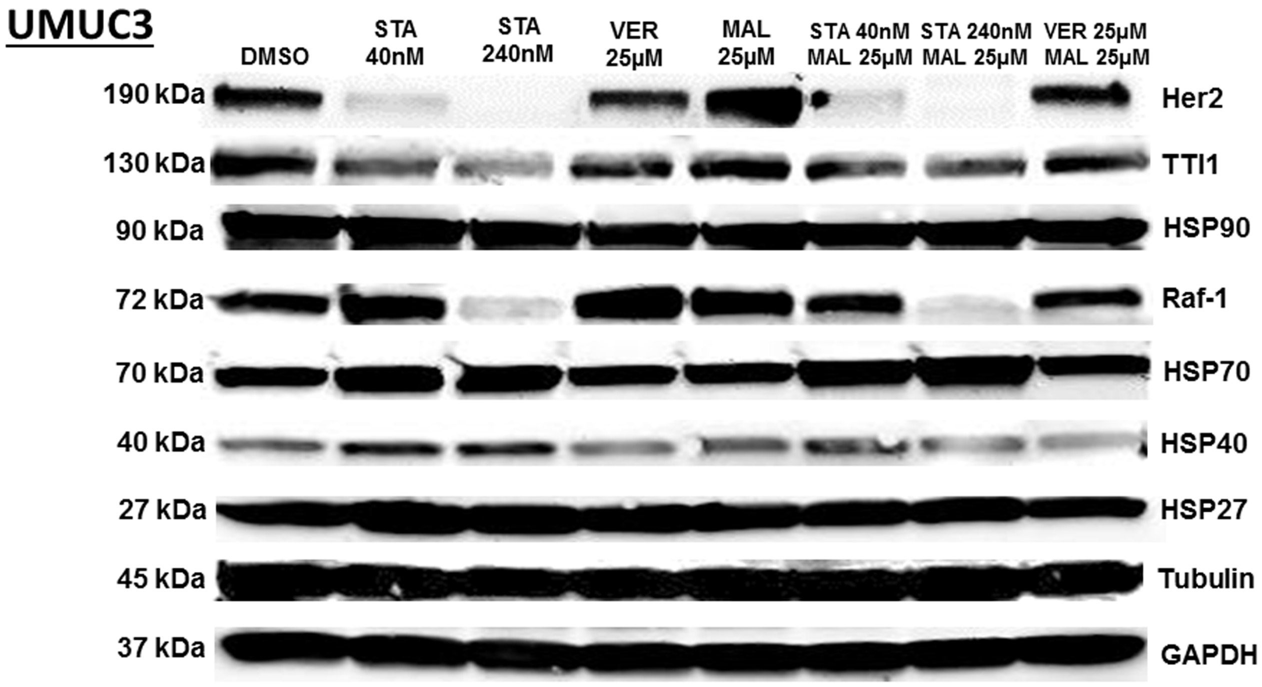

Supplementary Figure 11: HSP70 and HSP90 inhibitor single agents and combinations alter protein levels of critical kinases and HSPs at 48 hours. Drug doses that gave greatest reduction in cell viability or the most synergy were given to UMUC3 cells for 48 hours as single agents and in combination. Cells were lysed, followed by protein quantification and western-blot analysis.

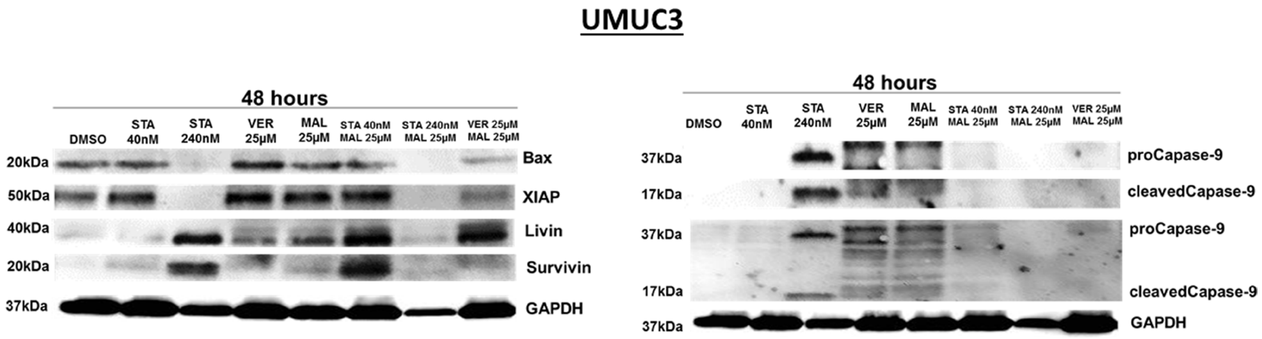

Supplementary Figure 12: HSP70 and HSP90 inhibitor single agents and combinations alter protein levels of apoptotic and pro-survival components at 48 hours. Drug doses that gave greatest reduction in cell viability or the most synergy were given to UMUC3 cells for 48 hours as single agents and in combination. Cells were lysed, followed by protein quantification and western-blot analysis. Total protein concentrations were normalized to the STA 240 nM + MAL 25 μM lane.

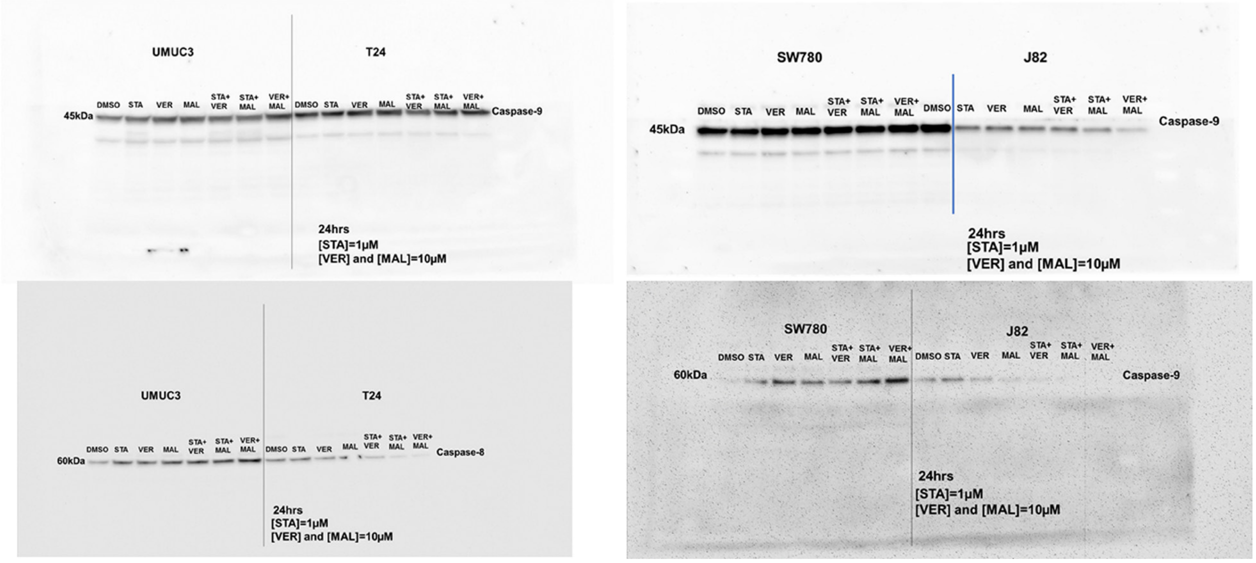

Supplementary Figure 13: HSP70 and HSP90 inhibitors do not significantly affect Caspase 9 and Caspase 8 cleavage. UMUC3, T24, SW780 and J82 cells were treated with either 1 µM STA, 10 µM VER, 10 µM MAL, 1 + 10 µM STA+VER, 1 + 10 µM STA+MAL or 10 + 10 µM VER+MAL for 24 hours. Cells were lysed, followed by protein quantification and western-blot analysis.

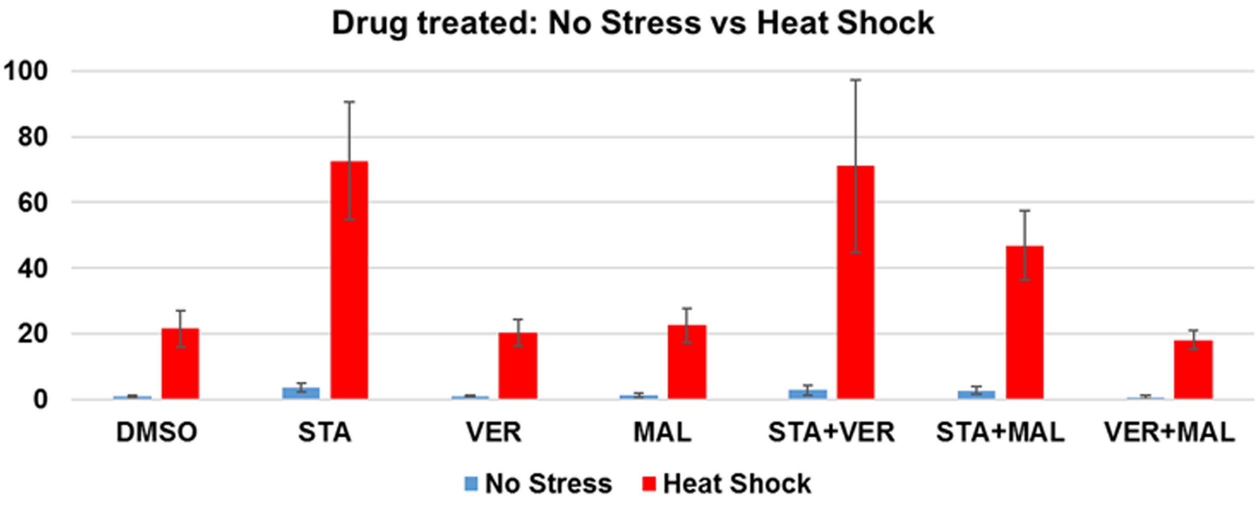

Supplementary Figure 14: HSP70 inhibitors do not increase the heat shock response. UMUC3 cells were transfected with a reporter plasmid encoding NanoLuc luciferase driven by a HSP70B' promoter. Cells were treated with the indicated drug concentrations for 9.5 hours then harvested and assayed for NanoLuc activity or were treated with the indicated drugs for 1 hour, heat shocked for 30 minutes at 45° C and allowed to recover for 8 hours before being harvested. NanoLuc activity for each condition was indexed to the DMSO no heat shock control.
